# Genomic surveillance reveals long-term endemicity and outbreak potential of Klebsiella pneumoniae sequence type 48 in a German hospital and its global context

**DOI:** 10.1099/mgen.0.001707

**Published:** 2026-07-20

**Authors:** Ngoc Minh Nguyen, Anna Weber, Basil Britto Xavier, Juan Pablo Rodríguez-Ruiz, Axel Kola, Michael Behnke, Christine Geffers, Herman Goossens, Stephan Harbarth, Marc Bonten, Rafael Canton, Petra Gastmeier, Youri Glupczynski, Friederike Maechler, Surbhi Malhotra-Kumar, on behalf of the R-GNOSIS WP5, STARCS WP2, and MISTAR WP1 study groups

**Affiliations:** 1 Laboratory of Medical Microbiology, Vaccine & Infectious Disease Institute, University of Antwerp, Antwerp, Belgium; 2 Institute of Hygiene and Environmental Medicine, Charité–Universitätsmedizin Berlin, Berlin, Germany; 3 Infection Control Program, Geneva University Hospitals and Medical School, Geneva, Switzerland; 4 Julius Center for Health Sciences and Primary Care, University Medical Center Utrecht, Utrecht University, Utrecht, Netherlands; 5 European Clinical Research Alliance on Infectious Diseases, Utrecht, Netherlands; 6 Servicio de Microbiología, Hospital Universitario Ramón y Cajal, Instituto Ramón Cajal de Investigación Sanitaria (IRYCIS), Madrid, Spain; 7 CIBER en Enfermedades Infecciosas (CIBERINFEC), Instituto de Salud Carlos III, Madrid, Spain

**Keywords:** antimicrobial resistance, international clone, *Klebsiella pneumoniae*, phylogenomics, sequence type ST48

## Abstract

*Klebsiella pneumoniae* sequence type 48 (Kp-ST48) is a globally distributed clone linked to antimicrobial resistance (AMR) yet lacks a comprehensive genomic analysis. Here, we investigated the persistence, transmission dynamics and global context of ST48 in a large tertiary hospital in Berlin, Germany. Between 2014 and 2022, 48 surveillance and 15 putative outbreak Kp-ST48 isolates were isolated in a tertiary care, multi-site hospital in Berlin, Germany. Genomic diversity was analysed by short- and long-read sequencing. Additionally, we included 223 publicly available Kp-ST48 genomes from five continents over 40 years (1982–2022) in the phylodynamic analysis. We identified two genetically distinct clades (A and B) within the global Kp-ST48 population. The global spread of Kp-ST48 was driven by clade B, which included all the genomes from the Berlin hospital. Two hospital-specific lineages (1 and 2) were identified with distinct population dynamics. Lineage 2 was transient and linked to a putative outbreak in 2019. Meanwhile, lineage 1 was first detected in 2014 and persisted for over 8 years until 2022, with multiple putative patient-to-patient and indirect transmission events identified. Carbapenem resistance determinants (*ompK35/36* mutations, *bla*_KPC_, *bla*_NDM_, *bla*_OXA-48_, *bla*_VIM_) were present in 57% (*n*=163/286) of genomes, and up to three *bla*_CTX-M-15_ copies were found integrated into chromosomes. Although Kp-ST48 generally did not contain a high number of virulence genes, 19 genomes showed potential for AMR-hypervirulence convergence. This study reveals the endemic persistence with outbreak potentials of Kp-ST48 in a hospital over 8 years, characterized by high genome plasticity. Our results highlight the global distribution of this clone, which warrants continuous surveillance.

**Impact Statement**

This study provides critical insights into the population structure and transmission dynamics of *Klebsiella pneumoniae* sequence type 48 (Kp-ST48), a globally disseminated, multidrug-resistant clone. By integrating genomic and epidemiological data over an 8 year period in a Berlin hospital, we reveal the sustained local endemicity and accumulation of carbapenem resistance within Kp-ST48. Our findings demonstrate that indirect transmissions, likely via undetected carriers or environmental reservoirs, played a key role in maintaining Kp-ST48 circulation, underscoring the importance of incorporating environmental and asymptomatic screening into infection control strategies. At the global scale, we delineate two major Kp-ST48 clades, identifying clade B as the primary driver of international dissemination. This work informs targeted interventions to prevent the emergence and spread of multidrug-resistant and potentially hypervirulent pathogens.

## Data Availability

The sequencing reads and assemblies of surveillance and outbreak isolates, respectively, are deposited in the SRA and assembly database in the National Center for Biotechnology Information and the European Nucleotide Archive under project numbers PRJNA548763 and PRJEB101711, respectively. All accession numbers of the 63 German genomes characterized in this study are included in the Additional file 1.xlsx.

## Introduction

Among the pathogens causing healthcare-associated infections, *Klebsiella pneumoniae* is the third most common nosocomial pathogen, causing severe infections and accounting for 10.4% of isolated micro-organisms [1]. In 2023, the estimated incidence of invasive *K. pneumoniae* in the European Union and European Economic Area was 24.2 cases per 100,000 population [2]. Nosocomial infections caused by *K. pneumoniae* are becoming difficult to treat, largely due to their multidrug resistance, hypervirulence or convergence of both traits [3,4].

In healthcare facilities, multidrug-resistant (MDR) *K. pneumoniae,* encountered within several high-risk clones, such as ST258, ST11 of CG258, ST15 and ST147, can spread rapidly but silently between patients and is a frequent cause of hospital outbreaks [5,6]. These clones are characterized by the high number of genes encoding resistance to multiple antibiotic classes, including last-resort antibiotics such as carbapenems and colistin [7]. In contrast, in community settings, hypervirulent clones, such as CG23, CG65 and CG86, are more common than resistance clones. These clones can cause life-threatening community-associated infections such as pyogenic liver abscesses, pneumonia, endophthalmitis or meningitis in healthy individuals [7]. Unlike hospital-associated MDR clones, these hypervirulent clones rarely carry antibiotic resistance genes (ARGs) but instead possess multiple virulence factors, including core capsules (K1 or K2), lipopolysaccharide (LPS O1 or O2) and acquired virulence factors (siderophore yersiniabactin, aerobactin, salmochelin, colibactin, *rmpA*, *rmpA2*), which contribute to their high pathogenicity [7]. Of increasing concern is the convergence of ARGs and hypervirulence genes, leading to the emergence of MDR hypervirulent clones [8,9]. For instance, *Klebsiella pneumoniae* carbapenemase (KPC)-producing CG258 isolates, mostly ST11 in China, have acquired yersiniabactin virulence genes. Conversely, hypervirulent clone ST23 carrying antimicrobial resistance (AMR) genes has been reported [8]. The most common convergence scenario observed so far is the acquisition of virulence plasmids or hybrid AMR-virulence plasmids in MDR clones, resulting in the enhanced pathogenicity of these hospital-associated MDR clones [7].

*K. pneumoniae* sequence type 48 (Kp-ST48), belonging to clonal group CG43 [10], has been recognised as a globally distributed MDR clone, described across six continents [10,23]. This clone has been associated with resistance to critical antibiotics, including carbapenems [24,26], third-generation cephalosporins [27,29], tigecycline [30] and colistin [31]. Although not a classical hypervirulent clone, carbapenem-resistant Kp-ST48 has been reported to harbour large virulence plasmids carrying acquired virulence factors such as siderophore systems and capsule up-regulation genes [14,19]. These MDR hypervirulent Kp-ST48 have been associated with hypervirulent infections, posing significant clinical concerns.

Kp-ST48 has been associated with multiple outbreaks in different healthcare settings including intensive care units (ICUs) and neonatal care units with mortality rate up to 30% [13,16, 20, 25]. During these outbreaks, Kp-ST48 was also recovered from hospital surfaces and fomites [16]. Beyond clinical samples, this clone was additionally identified in surveillance faecal samples from humans [32]. Notably, carbapenemase- and Extended-Spectrum Beta-Lactamase (ESBL)-producing Kp-ST48 have also been found in chicken meat [33] and wastewater from urban treatment plant [12], highlighting a potential zoonotic reservoir.

Despite multiple reports on the Kp-ST48 clone, these have been locally confined to case reports, and only limited studies have investigated the pangenome and population dynamics of this clone. Nevertheless, a comprehensive genomic analysis of this clone would provide insights into its evolution, population structure and niche adaptation. In this study, we report an endemic of the Kp-ST48 clone associated with putative carbapenem resistance outbreaks in a multi-site hospital in Berlin, Germany, over 8 years (2014–2022). Additionally, we investigate the global evolution and population diversity of Kp-ST48 using publicly available genomes from five continents collected over 40 years (1982–2022).

## Methods

### Settings and study design

We included in this study a set of *K. pneumoniae* ST48 from our two curated collections; both were collected from patients admitted to the Charité – Universitätsmedizin Berlin (Germany), a tertiary care centre with 3,011 beds located at three geographically separated sites. The first collection of 24 Kp-ST48 surveillance isolates was obtained during the EU – FP7-– funded project R-GNOSIS (Resistance of Gram-Negative Organisms: Studying Intervention Strategies, registration number ISRCTN57648070). These isolates were collected in two non-ICU wards of the hospital, as part of the two-arm, cluster-randomized, controlled clinical trial. The study design and protocol were described in detail elsewhere [34]. In brief, from 2014 to 2016, rectal swabs were obtained solely for study purposes from patients admitted to the wards at admission, weekly after that and at discharge. Chromogenic agar plates (BioMérieux, France) were used to identify ESBL-producing Enterobacterales (ESBL-E).

Outside the framework of R-GNOSIS, the second collection consisted of 15 Kp-ST48 genomes isolated from putative carbapenem resistance outbreaks occurring from 2019 to 2022. Additionally, 24 Kp-ST48 genomes were identified during retrospective surveillance. The majority of outbreak isolates (*n*=13) were from patients, with two environmental isolates collected from sinks.

No dedicated institutional review board approval was required as the samples were collected during a larger study and/or during routine infection prevention and control activities, which are mandated by German health authorities.

**Fig. 1.**
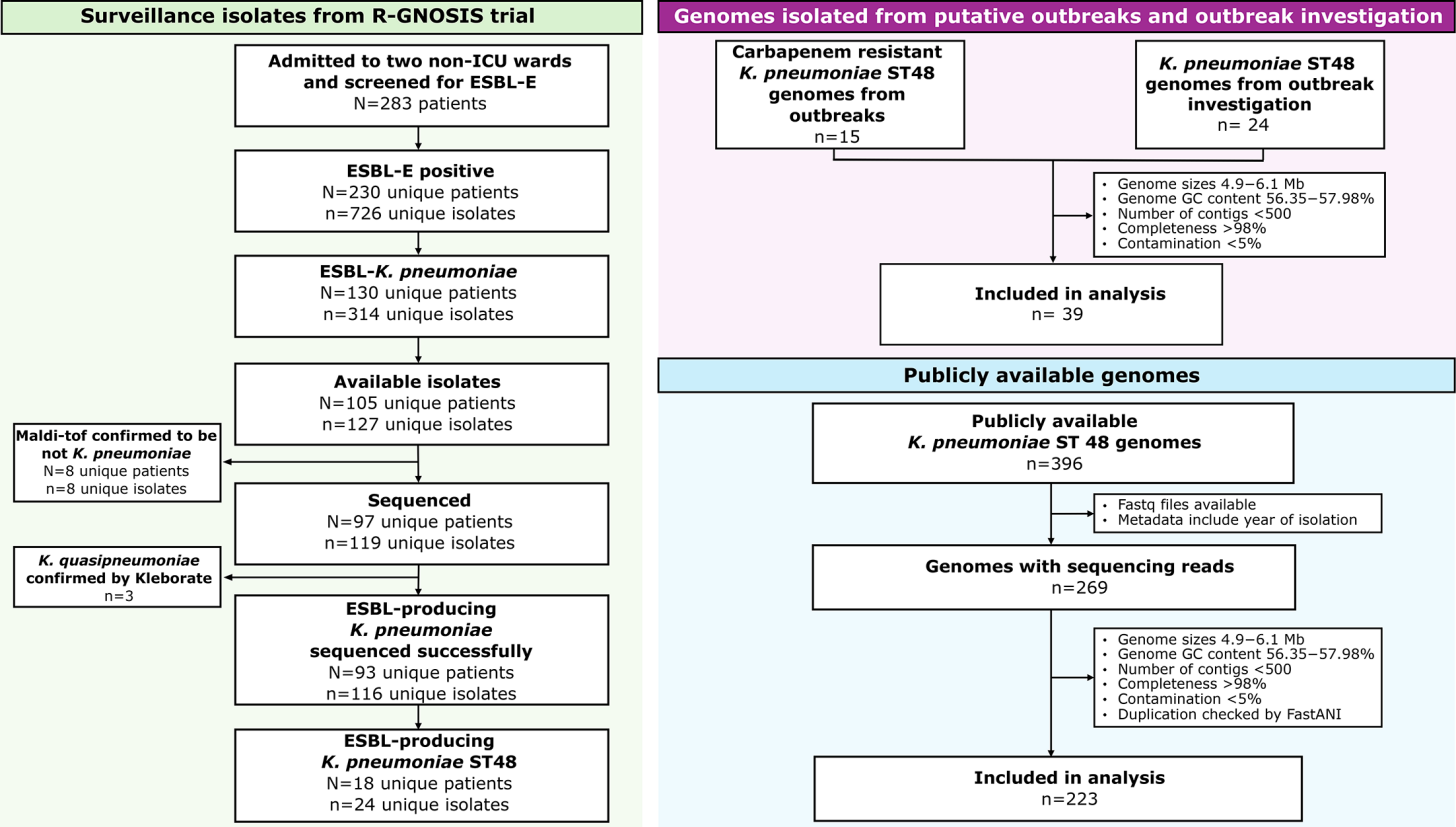
Surveillance isolates, putative outbreak and publicly available Kp-ST48 genomes included in this study. N: number of patients, n: number of isolates.

Additionally, we included global Kp-ST48 genomes from publicly available databases for phylogenetic and phylodynamic analysis. Of 396 Kp-ST48 genomes available in the Pathogenwatch repository, we included a total of 269 genomes with available raw sequencing reads and information on the isolation year for phylodynamic analysis (Fig. 1).

### Microbiological testing and whole-genome sequencing on surveillance isolates

Surveillance ESBL-E isolates were confirmed for species by MALDI-TOF (Bruker, Germany). Disc diffusion test (Rosco, Denmark) was used to confirm ESBL phenotype according to the CLSI M100-E34 guideline.

DNA from the isolated ESBL-producing *K. pneumoniae* (ESBL-Kp) was extracted using the MasterPure Complete DNA and RNA Purification kit (Epicentre, USA) and purified with the DNA Clean and ConcentratorTM-10 Kit (Zymo Research, USA). The library was prepared using the Nextera XT sample preparation kit, and 2×250 bp paired-end sequencing was performed using the MiSeq platform (Illumina Inc., USA).

Long-read sequencing on the PacBio Sequel platform (Pacific Biosciences, USA) was performed on four available ST48 ESBL-Kp isolates (P12D, P102D, P123A and P125A). Briefly, high-molecular-weight (HMW) genomic DNA from these isolates was extracted using MagAttract HMW DNA extraction kit (Qiagen, Germany), the SMRT library was prepared using SMRTbell Template Prep kit 1.0 (Pacific Biosciences, USA) and sequencing was performed on Sequel with 10 h movie time using single-molecule real-time (SMRTCell v3) 1M (Pacific Biosciences, USA). The SMRT Analysis portal version 7.0 was used for de-multiplexing and filtering the reads with default parameters. The *de novo* assembly was performed using the Hierarchical Genome Assembly Process, which was available through the SMRT Analysis portal version 7.0. The generated assemblies were further polished using Pilon v1.23 [35] and Polypolish 0.6.0 [36].

### Whole-genome sequencing data analysis

Low-quality and adapters were trimmed with Trim Galore v0.6.4; subsequently, raw reads from short-read sequencing were *de novo* assembled using SPAdes v3.15.0 [37]. Assembly quality was evaluated using Quast v5.0.2 [38] and checkm v1.1.2 [39]. Genomes with sizes 4.9–6.1 Mb, G+C content 56.35–57.98 mol%, <5% contamination, >98% completeness and assemblies of less than 500 contigs were included in further analysis.

Kleborate v2.3.2 [40,41] was used to confirm *K. pneumoniae* identification within the *K. pneumoniae* species complex, identify sequence types (STs), resistance genes and virulence factors. Virulence score and resistance score were also computed within Kleborate; AMR-virulence convergence was defined as a virulence score ≥3 and a resistance score ≥1 [4]. Plasmid replicons were typed using PlasmidFinder v2.1.6 [42]. To investigate the potential presence of hypervirulence plasmids, a collection of 79 well-characterized hypervirulence plasmids [43] was downloaded from NCBI and used as reference genomes for read mapping using bowtie2 v2.5.4 [44] with a minimum read depth of 10×. Statistics of mapping were generated by samtools v1.7 [45].

Genome annotation was performed using Prokka v1.14.5 [46]. The resulting gff files were subjected to pangenome analysis using Roary v3.13.0. Insertion sequence (IS) elements and prophage sequences were identified using the ISfinder [47] and PHASTEST [48], respectively. Default thresholds were used for these typing analyses unless mentioned otherwise.

Whole-genome core-SNP alignment of the 286 ST48 genomes was constructed by snippy v4.6.0 [49]. The long-read whole-genome sequence of the earliest surveillance isolate (P12D), which carried no plasmid, was used as the reference. SNP distance was calculated by snp-dists v0.7.0 [50]. The recombination regions were detected from the alignment using Gubbins v2.4.1 [51]. Phylogenetic trees were constructed using the recombinant-free whole-genome core-SNP alignment using iqtree v2.2.5 [52], with 1,000 bootstrap replicates. The generated tree was visualized using iTOL v.7 [53]. Large genomic rearrangements were identified and visualized using pyGenomeViz v1.1.0 [54].

### Bayesian phylodynamic analysis

The dated phylogeny was inferred by BactDating v1.1.2 [55] using the recombination-corrected phylogeny. The years of isolation were used as the tip dates. Separated Markov chain Monte Carlo (MCMC) chains were run for 1e5 and 1e7 iterations under all clock models. Bayesian comparison between models based on the deviance information criterion (DIC) values was computed within BactDating. To test the temporal signal of the dataset, the MCMC chains were rerun under the assumption that all genomes were sampled in the same year of 2000. The best-fit model was the model with the smallest DIC value, and convergence was confirmed using the Gelman–Rubin diagnostic on three independent chains, with potential scale reduction factor values close to 1.

A MCMC chain of 1e8 iterations was performed under the best-fit model. Proper convergence and mixing properties were determined by assessing the traces and the effective sample size (ESS) for the inferred parameters at the threshold of 200. The ESS test was implemented using the R package coda v0.19-4.1 [56]. R packages were implemented in R v.4.3.1. The time tree was visualized using FigTree v.1.4.4 and annotated using iTol v.7 [53].

The time-scaled phylogeny for 63 surveillance and outbreak genomes collected from the studied hospital was inferred using BactDating under the Poisson strict clock model with a 1e7-iteration MCMC chain. The timed tree was used as input for the R package TransPhylo v1.4.10 [57] to infer putative transmission events and reconstruct the transmission tree. The gamma distribution with a shape parameter of 1.2 and a scaling parameter of 1.0, as previously reported [58], was used as a prior for the generation-time distribution. The MCMC was run for 1e5, 1e6 and 1e7 iterations, and the ESS threshold was set as 100 to ensure proper mixing and MCMC convergence.

## Results

### Endemic ESBL-producing Kp-ST48 in the hospital setting

Within the R-GNOSIS study (2014–2016), from 230 included patients, 726 surveillance ESBL-E isolates were collected. A total of 314 (43.3%) unique isolates (from 130 patients) were identified as ESBL-Kp isolates (Fig. 1). Of these, 127 unique isolates were available for species confirmation, and 119 (37.9%, from 97 patients) were successfully sequenced (Fig. 1). Kleborate identified three genomes belonging to *Klebsiella quasipneumoniae* subsp*. quasipneumoniae*, which were excluded, and thus 116 genomes were further analysed. Of these, 24 (20.7%) were identified as Kp-ST48, making it the most dominant ST, followed by ST15 (9/116, 7.8%) and ST307 (8/116, 6.9%).

Outside the framework of R-GNOSIS, between 2019 and 2022, 15 carbapenemase-producing Kp-ST48 were isolated from patients (*n*=13) and the environment (sinks, *n*=2), representing an increase above the baseline incidence (0%) and indicating putative outbreaks. Nine, five and one carbapenem-resistant Kp-ST48 isolates were collected in 2019, 2020 and 2022, respectively, from the ICU and non-ICU wards of one hospital site. During retrospective surveillance between 2014 and 2022, 24 screening Kp-ST48 were identified from patients in various ICU and non-ICU wards across three hospital sites, including the site with Kp-ST48 putative outbreaks.

Among the publicly available Kp-ST48 genomes, a total of 223 genomes passed the post-assembly QC check and were included in further analysis (Fig. 1). These genomes were collected from 32 countries on five continents from 1982 to 2022 (Table 1). Humans were the main hosts (220/223, 98.7%); one genome was isolated from a dog, and two were isolated from unknown hosts. Accession numbers and characteristics of these genomes are detailed in Supplementary Material 1.

**Table 1.** Geographic distribution of the publicly available Kp-ST48 genome collection across continents and countries

Clade	Continent	n (% within clade)		Country	n (% within clade)		BioProject accession
Clade A, *n*=37	Africa	1	2.7%	Algeria	1	2.7%	PRJNA672836
	Asia	5	13.5%	Singapore	4	10.8%	PRJNA547865
							PRJNA577535
				Thailand	1	2.7%	PRJDB5929
	Europe	2	5.4%	United Kingdom	2	5.4%	PRJNA788733
	North America	28	75.7%	USA	27	73.0%	PRJNA288601
							PRJNA339843
							PRJNA396774
							PRJNA433394
							PRJNA496461
							PRJNA633565
							PRJNA658369
							PRJNA686897
							PRJNA803478
				Canada	1	2.7%	PRJNA855907
	Oceania	1	2.7%	New Zealand	1	2.7%	PRJNA398288
Clade B, *n*=249	Africa	46	30.9%	South Africa	20	8.0%	PRJEB46655
							PRJEB63361
				Nigeria	9	3.6%	PRJEB33565
							PRJNA351846
				Kenya	5	2.0%	PRJNA804332
				Madagascar	4	1.6%	PRJEB29143
				Rwanda	3	1.2%	PRJEB33565
				Ghana	2	0.8%	PRJEB37523
							PRJNA473419
				Congo	2	0.8%	PRJEB56212
				Tanzania	1	0.4%	PRJNA503964
	Asia	67	26.9%	Bangladesh	44	17.7%	PRJEB39293
							PRJNA845975
				Pakistan	5	2.0%	PRJEB39293
							PRJNA308116
				Singapore	3	1.2%	PRJEB13304
							PRJNA577535
				Thailand	3	1.2%	PRJDB5929
							PRJEB38540
							PRJNA389557
				Iran	3	1.2%	PRJEB59975
				India	2	0.8%	PRJEB30913
							PRJNA548120
				China	2	0.8%	PRJNA761884
							PRJNA956822
				Qatar	2	0.8%	PRJNA599387
				Saudi Arabia	2	0.8%	PRJEB36683
				Cambodia	1	0.4%	PRJEB29143
	Europe	125	50.2%	Germany	72	28.9%	PRJNA548763/PRJEB101711 (this study)
							PRJEB18059
							PRJEB39867
				United Kingdom	33	13.3%	PRJEB48990
							PRJEB5065
							PRJNA564424
							PRJNA788733
				Norway	8	3.2%	PRJEB27256
							PRJEB42350
				Ireland	5	2.0%	PRJEB19229
				Switzerland	2	0.8%	PRJEB28660
							PRJNA543274
				Netherlands	2	0.8%	PRJEB35685
							PRJNA754858
				France	1	0.4%	PRJNA675776
				Spain	1	0.4%	PRJEB63349
				Portugal	1	0.4%	PRJEB38289
	North America	2	0.8%	USA	1	0.4%	PRJNA288601
				Canada	1	0.4%	PRJNA564992
	Oceania	9	3.6%	Australia	9	3.6%	PRJEB2111
							PRJNA529744
							PRJNA797179

The ST48 genomes, from both the studied hospital and public database, had a median genome size of 5,502,810 bp (range: 5,087,132–5,926,149 bp) and an average G+C content of 57.25 mol% (range: 56.6–57.7 mol%).

### Phylogenomics reveals the national and international distribution of Kp-ST48

**Fig. 2.**
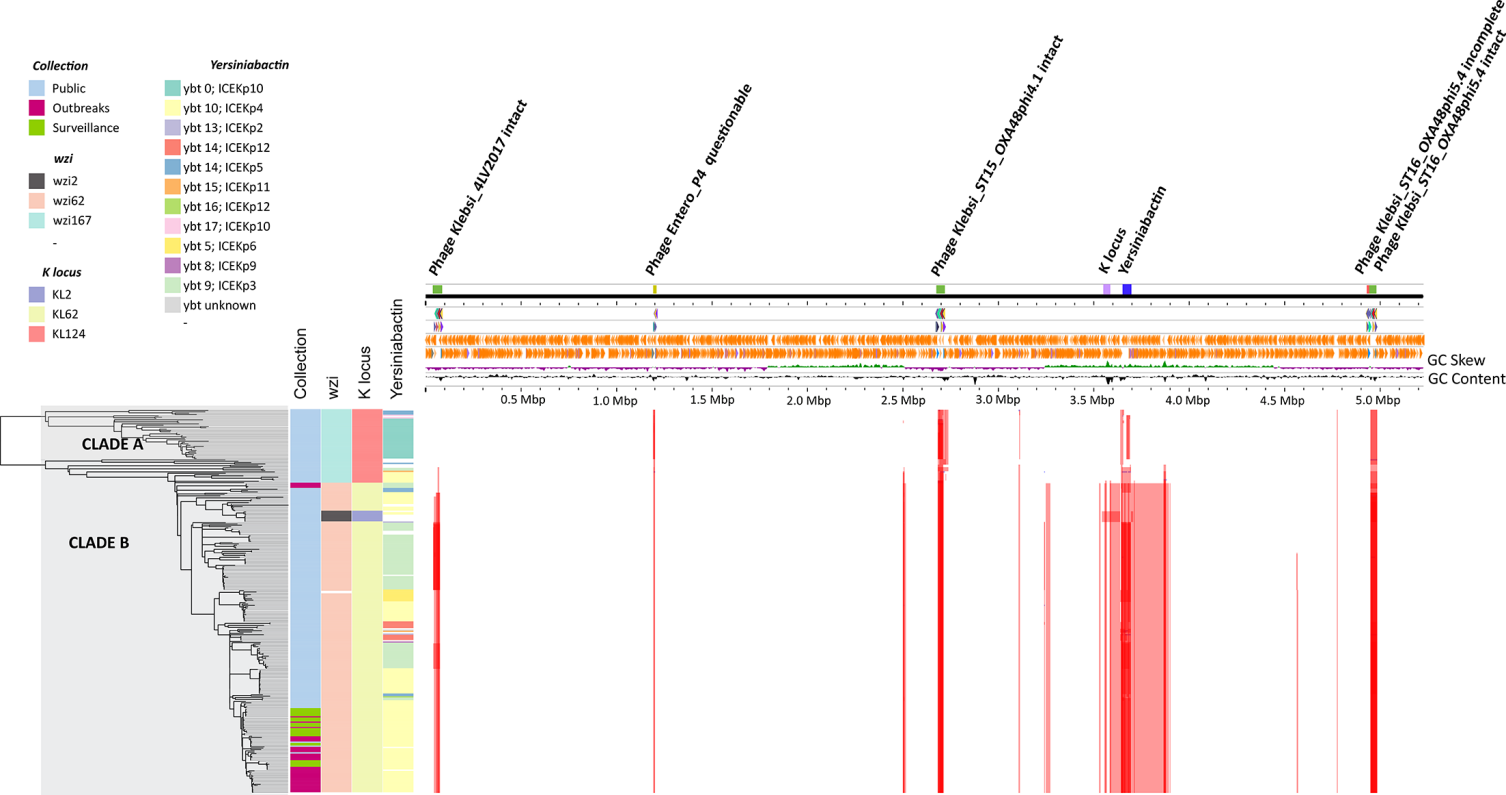
Phandago plot of recombinations detected with Gubbins across maximum likelihood phylogeny of Kp-ST48 clone. PacBio-sequenced P12D genome was used as the reference. Phages were determined using Phastest.

Phylogenomic analysis revealed two genetically distinct clades of Kp-ST48, labelled as clade A (37/286, 12.9%) and B (249/286, 87.1%, Fig. 2). Clade A comprised 37 genomes isolated from 2013 until 2022 from seven countries, mainly from the USA (27/37, 73.0%, Table 1). Clade B consisted of 249 genomes from 30 countries that distinctively diverged from clade A by recombination events involving 375 kb of phage, the K locus and yersiniabactin coding regions (Fig. 2). Excluding the 63 genomes collected from the studied hospital, which were clonally related and thus biased, clade B was mainly identified from Bangladesh (44/249, 17.7%), the UK (33/249, 13.3%) and South Africa (20/249, 8.0%, Table 1). Clade B accommodated not only genomes isolated from humans but also from the environment (sinks, *n*=2, this study) and from one dog host in the public database.

**Fig. 3.**
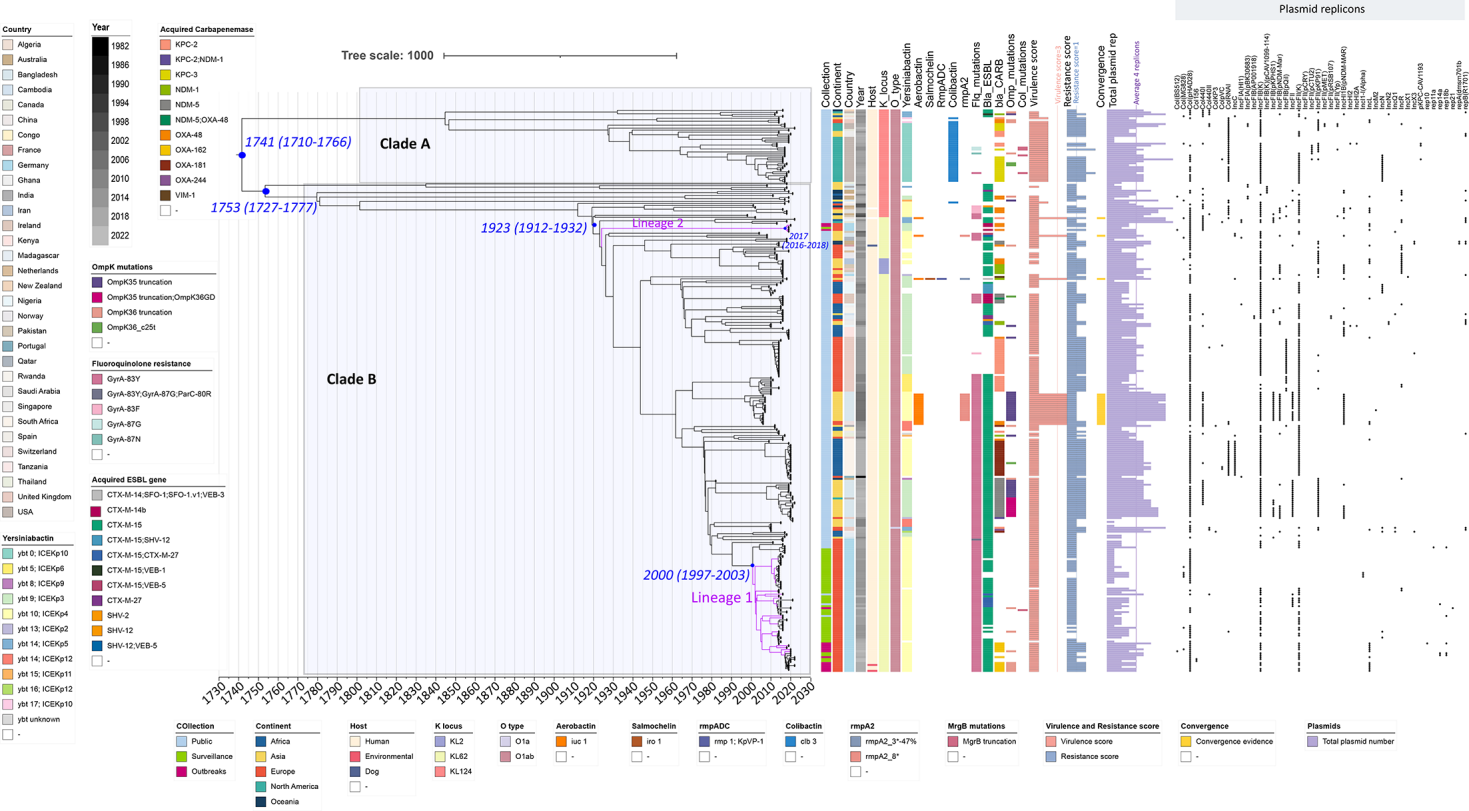
Evolutionary reconstruction of the Kp-ST48 population with two clades, A and B, and two hospital lineages, 1 and 2. A time-scaled tree was inferred from the recombination-corrected phylogeny using BactDating. The x-axis represents the emergence time estimates.

The two clades A and B differed from each other by 621 non-recombinant pairwise SNPs on average (range 463–763). Within clade B, the genomes collected from the studied hospital over 8 years (2014–2022) clustered into two monophyletic lineages (1 and 2) with a bootstrap support of 100%, suggesting hospital-specific lineages (Fig. 3).

### Genetic determinants of pathogenicity and resistance in Kp-ST48 show potential of AMR-virulence convergence

**Fig. 4.**
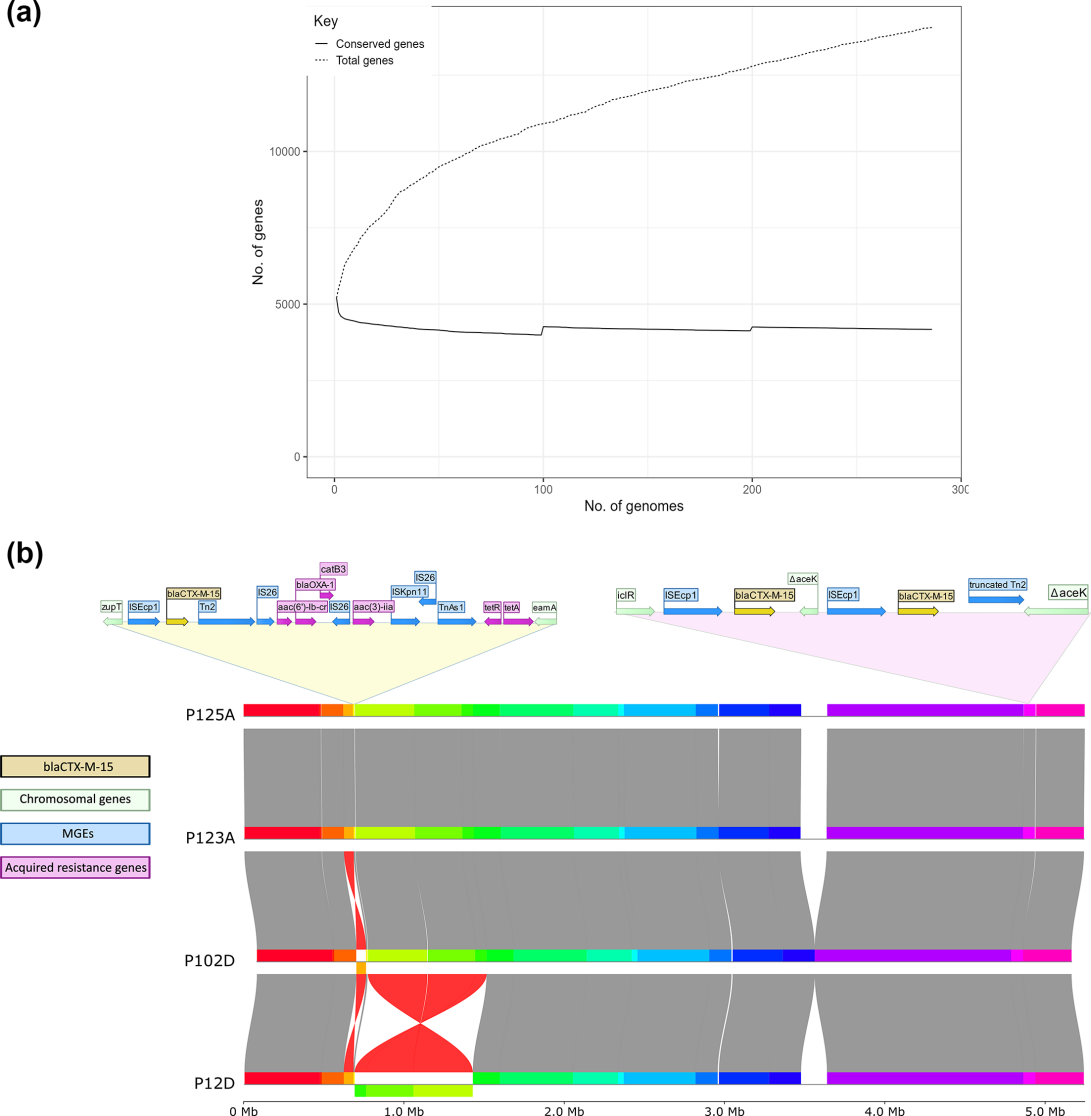
(**a**) Pan-genome plot of 286 *K*. *pneumoniae* ST48 genomes indicating an open genome with the number of genes increased when new genomes were added. (**b**) Genome rearrangement with chromosome synteny, inversions and chromosomal integration of *bla*_CTX-M-15_ observed among surveillance genomes long-read sequenced with PacBio.

The pan-genome analysis revealed an open genome with 14,058 genes, of which 4,172 (29.7%) were core genes (present in ≥99% strains, Fig. 4a). The core genome was ~3.9 Mbp in size. We observed chromosome inversions of ~60 kbp and 736 kbp regions between the four complete chromosomes of the surveillance isolates, which were isolated 8–185 days apart, highlighting the genomic plasticity mediated by chromosomal rearrangement (Fig. 4b).

Kp-ST48 did not exhibit a high diversity of capsule polysaccharide K antigen types nor hypervirulence-associated types (e.g. K1, K2, K5, K20, K54, K57), except for K2, which was identified in eight genomes from the public database. We observed capsule locus switching with capsular synthesis loci (K-loci) specific for clades and sub-clades. Three K-loci correlated with three *wzi* alleles were identified: KL2, KL62 and KL124. Of these, KL62 (*wzi62*, 223/286, 78.0%) was the most prevalent and presented only in clade B (Fig. 3). KL124 (*wzi167*) was identified in 19.2% (55/286) of Kp-ST48 genomes, most predominant in clade A (*n*=47). Meanwhile, 2.8% (8/286) of genomes were associated with KL2 (*wzi2*), distinctively identified in a monophyletic lineage of clade B (Fig. 3). For the O locus, the O1/O2v1 allele of the LPS O antigen was identified in all genomes (Fig. 3).

In contrast, the siderophore yersiniabactin exhibited greater diversity with 11 yersiniabactin loci (*ybt*), including a novel type, and associated with 9 integrative conjugative elements (ICE*Kp*). In clade A, *ybt*0, typically mobilized by ICE*Kp10*, was predominant (30/37, 81%), whereas in clade B, *ybt*10 associated with ICE*Kp4* (122/249, 49%) was the most prevalent type across continents, followed by *ybt*9-ICE*Kp3* (73/249, 29%, Fig. 3).

Aerobactin (*iuc*, *n*=19) and salmochelin (*iro*, *n*=1) loci were rarely present (Fig. 3), detected together in only one genome isolated from China in 2015, which also carried the only *rmp*1 locus associated with IncFIBk virulence plasmid KpVP-1. Genotoxin colibactin was infrequent, with *clb*3 present in 33 genomes, mostly within clade A (Fig. 3). The genomes from the studied hospital show no evidence of hypervirulence characteristics, except for yersiniabactins.

Regarding resistance genotypes, besides the intrinsic *fosA*, *oqxAB* and *bla*_SHV_ genes, we identified on average 10 (range: 0–24) acquired ARGs (Additional file 1). These genes were associated with resistance to aminoglycosides (255/286, 89.2%), sulfonamide (232/286, 81.1%), tetracyclines (189/286, 66.1%), trimethoprim (178/286, 62.2%), macrolides (151/286, 52.8%), fluoroquinolones (86/286, 30.1%), phenicols (60/286, 21.0%) and rifampicin (16/286, 5.6%). Chromosomal mutations, in gyrase GyrA (Ser83Phe, Ser83Tyr, Asp87Gly, Asp87Asn) and topoisomerase ParC (Ser80Arg), conferring fluoroquinolone resistance were identified in 172 (60.1%) genomes (Fig. 3).

A total of 214 (74.8%) genomes carried acquired ESBL genes, the majority being *bla*_CTX-M-15_ (198/286, 69.2%). This gene was identified across all clades, either alone or in combination with other ESBL genes (*bla*_CTX-M-14_, *bla*_CTX-M-27_, *bla*_SHV-2_ and *bla*_SHV-12_). Four isolates from lineage 1 were subjected to long-read sequencing, revealing the presence of multiple and up to three copies of *bla*_CTX-M-15_ integrated into the chromosomes (Fig. 4b).

Carbapenem resistance determinants of both carbapenemase-encoding genes (142/286, 49.7%) and outer membrane porins OmpK35/36 mutations (59/286, 20.6%) were identified. Variants of *bla*_KPC_, *bla*_NDM_, *bla*_OXA-48_ and *bla*_VIM_ were recorded, with *bla*_KPC-2_ being the most common (34/286, 11.9%, Fig. 3, Additional file 1). The distribution of these variants varied across geographic regions (continent/most dominant variant): Africa/*bla*_OXA-181_ (19/20, 95%), Asia/*bla*_NDM-5_ (18/39, 46.2%), Europe/*bla*_KPC-2_ (26/57, 45.6%), North America/*bla*_KPC-3_ (18/21, 85.7%) and Oceania/*bla*_NDM-5_ (3/5, 60%).

The genomes isolated from the studied hospital predominantly carried *bla*_CTX-M-15_ (50/63, 79.4%) across the study period (2014–2022). Of these, 14, isolated from 2019 onwards, additionally harboured *bla*_OXA-48_/*bla*_OXA-162_ and IncL plasmid replicons. Mapping to the IncL/M plasmid pOXA-48 (JN626286.1) showed 94 and 100% coverage at 10× minimum depth for *bla*_OXA-48_ and *bla*_OXA-162_, respectively. The OXA-162, which differs from OXA-48 by one amino acid (Thr213Ala), was found in hospital-specific lineage 1, whereas *bla*_OXA-48_ was associated with lineage 2. Notably, the loss and truncations of *mgrB* gene, associated with colistin resistance, were detected in five genomes, four of which were from clade A (Fig. 3, Additional file 1).

Overall, Kp-ST48 did not carry an extensive number of virulence factors with low virulence score. The majority (73.1%) of the genomes have virulence score of 1 (carried only *ybt*, *n*=209/286). Thirty-three genomes (11.5%) have virulence score of 2 (carried *clb*, with or without *ybt*). However, a high prevalence of Kp-ST48 carried genes conferring resistance to clinically important antibiotics including ESBL (*n*=119/286, 41.6%, resistance score=1), carbapenem (*n*=140/286, 49.0%, resistance score=2) and a combination of carbapenem and colistin (*n*=2/286, 0.7%, resistance score=3). Importantly, 19 genomes showed convergence potential, with all carrying aerobactin *iuc*1 loci and all but one carrying carbapenem resistance determinants (*bla*_NDM-1_, *bla*_OXA-48_, *bla*_KPC-2_ or OmpK mutations). These genomes were isolated from Bangladesh (*n*=15), China (*n*=2) and Switzerland (*n*=2). Bangladeshi genomes were isolated in one project (PRJNA845975, 2006–2008), likely linked to an outbreak, and showed 72–85% coverage of hypervirulence plasmids (MK181633.1 and CP040595.1, Additional file 1).

### Bayesian phylogenetic analysis reveals two introductions of Kp-ST48 into the hospital, with epidemiology-supported transmission links

Among the clock models tested, the Poisson strict model was best supported by the data upon comparison of DIC values (Table S1, available in the online Supplementary Material) and the Gelman–Rubin convergence diagnostic (Fig. S1). The mean evolution rate was estimated at 1.36 substitutions genome^−1^ year^−1^ (95% confidence intervals [CI]: 1.24–1.49), corresponding to 2.47×10^−7^ substitutions site^−1^ year^−1^ (95% CI: 2.25×10^−7^ −2.71×10^−7^). The first divergence event that separated the ST48 population into clade A and clade B occurred in 1741 (95% CI: 1710–1766, Fig. 3).

The time-tree phylogeny suggested that Kp-ST48 may have been introduced into the Berlin hospital in two distinct events, forming two lineages. Lineage 1 emerged as early as 2000 (95% CI: 1997–2003), was first detected in the hospital in 2014, persisted throughout the study period until 2022, and included 59 isolates from 51 patients. Meanwhile, lineage 2 emerged in 2017 (95% CI: 2016–2018) with four isolates from four patients collected in 2019. Despite the recent introduction and carrying *bla*_OXA-48_, lineage 2 did not persist over the years nor show any evidence of replacing lineage 1. Three publicly available genomes from humans in Germany, outside of the studied hospital, were interspersed within lineage 1 alongside our surveillance and outbreak genomes (Fig. 3), indicating transmission across Germany.

**Fig. 5.**
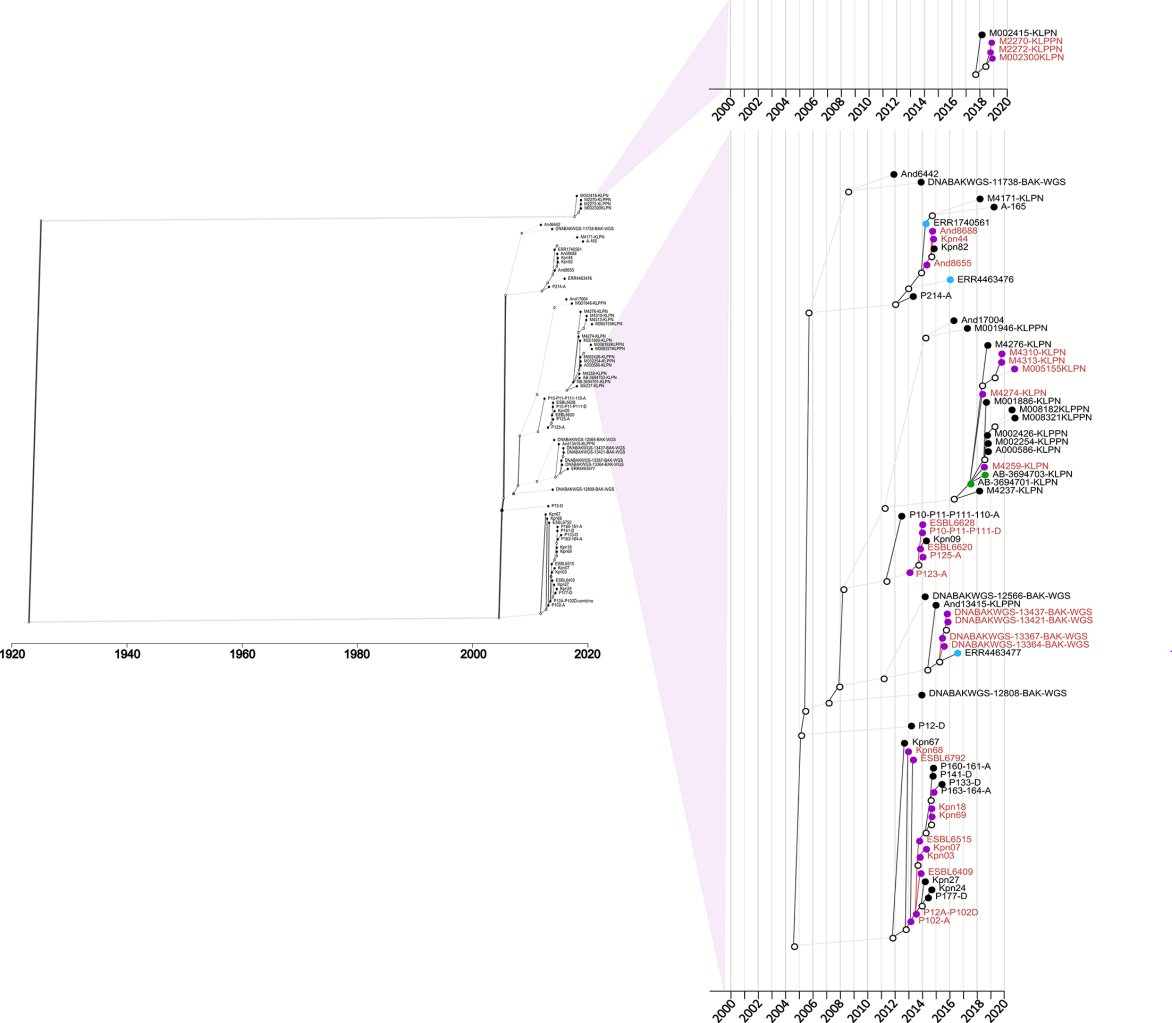
Transmission tree inferred transmission events. Each case is represented as a circle: a filled circle represents a sampled case, whereas an empty circle represents an unsampled case. Filled circles in purple represent isolates involved in transmission links supported by epidemiological links. Blue- and green-filled circles represent genomes from the public database and environmental isolates from hospital sinks, respectively. A link from one circle to another represents a transmission with direction from left to right. The x-axis shows the timeline when the genomes were isolated.

Using the average substitution rate of 1.36 genome^−1^ year^−1^, and accounting for isolation times, we identified 47 potential transmission links involving 37 genomes, differing by 0–5 SNPs over 0–6 years. Of these, 33 links involving 31 genomes were epidemiologically supported by overlapping hospitalization stays between patients, either in the same rooms (*n*=22 links) or same wards (*n*=11 links, Fig. 5). TransPhylo inferred a transmission tree among the local Kp-ST48 genomes with good convergence and mixing MCMC properties (Fig. S2). Among 22 transmission links between roommate patients, the transmission tree inferred 6 direct patient-to-patient transmission events, 2 indirect transmissions involving an intermediary patient and environmental source (sink) and 14 indirect transmissions attributed to unsampled sources. Of 11 transmissions between patients on the same wards, 1 was direct, while the remaining involved unsampled sources (Fig. 5).

## Discussion

This study identifies an endemic persistence of Kp-ST48 in a multi-site hospital setting in Germany, and its association with putative outbreaks, and further determines the international population structure of this clone. We observed a high genome plasticity within this clone, a critical trait that enables *K. pneumoniae* to acquire new genetic material [59]. This is evidenced by extensive recombination events, genome rearrangements, the acquisition of putative plasmids carrying virulence factors and carbapenemase-encoding genes *bla*_OXA-48/162_. Additionally, the chromosomal integration of *bla*_CTX-M-15_, which could be beneficial for stable propagation [60], may explain the persistence of this clone in the studied hospital.

The continuous isolation of Kp-ST48, conferring resistance to ESBL and carbapenems during surveillance and putative outbreaks within the hospital over 8 years (2014–2022), indicated an endemic presence of the Kp-ST48 clone. The presence of Kp-ST48 across geographically distant hospital sites was likely due to the transfer of patients (and/or healthcare staff) between sites. Two hospital-specific lineages with distinct epidemiological dynamics were observed. While lineage 2 emerged more recently with transient presence in 2019, lineage 1 persistently circulated over 8 years, during which it acquired *bla*_OXA-48/162_-carrying plasmids. Furthermore, the presence of three German genomes from the public database within the hospital-specific lineage 1 suggested that the spread of this lineage extended beyond a single hospital setting.

The mean evolution rate of Kp-ST48 was estimated at 1.36 substitutions genome^−1^ year^−1^ or 2.47×10^−7^ substitutions site^−1^ year^−1^, comparable to that of other MDR high-risk clones such as CG258 (2.99×10^−7^ substitutions^−1^ site^−1^ year) [61], but lower than the rates estimated for carbapenem-resistant *K. pneumoniae* ST11 (4.85 substitutions genome^−1^ year^−1^) [62] and the hypervirulent CG23 (3.40×10^−7^ substitutions site^−1^ year^−1^) [63].

Although Kp-ST48 is not typically a highly virulent clone, this clone holds the potential of AMR-hypervirulent convergence, demonstrated in 19 genomes across three countries harbouring carbapenem resistance and aerobactin virulence factor, which is associated with hypervirulent *K. pneumoniae* [64].

Our findings highlight that while a minority of Kp-ST48 transmission events occurred through direct patient-to-patient contact in shared hospital rooms, the majority were likely mediated by indirect routes, including undetected carriers (such as patients, healthcare workers, visitors) or environmental reservoirs. This underscores the role of asymptomatic carriage and unsampled sources in sustaining transmission within the hospital. The genetic linkage between environmental (sink) samples and patient isolates highlights the potential role of hospital sinks as persistent sources of transmission. Overall, this underscores the importance of addressing both direct contact and environmental contamination in infection prevention strategies. The identification of Kp-ST48 from hospital sinks corroborates other studies reporting the presence of this clone in environmental sources [12].

A potential limitation of our study lies in the collection of Kp-ST48 genomes. The surveillance genomes were selected based on ESBL phenotype, which may have led to an underestimation of the prevalence of the Kp-ST48 clone in the studied hospital. Additionally, the use of publicly available genomes could have introduced a bias towards closely related genomes from outbreaks and certain phenotypes. Furthermore, we were unable to identify any genomic differences between lineage 1 and lineage 2 that might account for the persistence of lineage 1 and the transient existence of lineage 2. Despite these limitations, our findings provide valuable insights into the epidemiology, population dynamics and evolution of this international clone.

In conclusion, our study provides a comprehensive understanding of the population structure and evolution of the globally distributed *K. pneumoniae* ST48 clone. We identified genetic markers associated with Kp-ST48 clades and highlighted the potential of causing outbreaks and AMR-hypervirulence convergence of this clone.

## Supplementary material

10.1099/mgen.0.001707 Supplementary Material 1.

10.1099/mgen.0.001707 Supplementary Material 2.

## Abbreviations

AMR: antimicrobial resistance
ARG: antibiotic resistance gene
CG: Clonal group
DIC: deviance information criterion
ESBL: Extended-Spectrum Beta-Lactamase
ESBL-Kp: ESBL-producing Klebsiella pneumoniae
ESS: effective sample size
HMW: high molecular weight
ICE: integrative conjugative element
ICU: intensive care unit
IS: insertion sequence
KPC: Klebsiella pneumoniae carbapenemase
Kp-ST48: *Klebsiella pneumoniae* sequence type 48
LPS: lipopolysaccharide
MCMC: Markov chain Monte Carlo
MDR: multidrug resistant
R-GNOSIS: Resistance of Gram-Negative Organisms: Studying Intervention Strategies
ST: sequence type
