# Supplementary material for: Genomic surveillance reveals long-term endemicity and outbreak potential of Klebsiella pneumoniae sequence type 48 in a German hospital and its global context
Source: Microb Genom. 2026 Jul 20;12(7):001707. doi: 10.1099/mgen.0.001707 (PMC13384113; doi:10.1099/mgen.0.001707)
Supplement: Supplementary Material 1. [file mgen-12-01707-s001.pdf]

Supplementary material

This supplementary material includes supporting information alongside the article "*Genomic surveillance reveals long-term endemicity and outbreak potential of Klebsiella pneumoniae sequence type 48 in a German hospital and its global context*".

Supplementary Table S1. *Clock model testing for Bayesian phylogenetic analysis using BactDating with 1e5 and 1e7 iterations of MCMC chains. The DIC values of Poisson model across the two chains of 1e5 and 1e7 iterations and in comparison with other models indicate a considerable temporal signal and best fit.*

	**Model**	**MCMC chains of 1e5 iterations**			**MCMC chains of 1e7 iterations**		
		**Mean substitution rate (µ)**	**95% CI**	**DIC**	**Mean substitution rate (µ)**	**95% CI**	**DIC**
**arc**	With temporal signal	1.95E+00	1.69e+00−2.27e+00	3618	1.96e+00	1.64e+00;2.36e+00	3706.41
	No temporal signal	8.67E-01	6.66e-01−1.21e+00	3318.28	1.08e-01	9.14e-03;3.94e-01	5950.2
**negbin**	With temporal signal	3.20E+00	2.52e+00−4.03e+00	4503.91	3.07e+00	2.33e+00;3.99e+00	4357.89
	No temporal signal	1.44E-01	9.19e-02−2.51e-01	4164.79	8.00e+00	2.26e-01;2.67e+01	5684.09
**poisson**	With temporal signal	1.48E+00	1.41e+00−1.55e+00	3482.94	1.36e+00	1.23e+00;1.50e+00	3550.61
	No temporal signal	9.39E-01	8.42e-01−1.01e+00	3619.1	9.60e-01	3.19e-01;1.93e+00	4109.79
**carc**	With temporal signal	1.94E+00	1.44e+00−2.33e+00	3643.43	2.02e+00	1.69e+00;2.39e+00	3746.71
	No temporal signal	8.62E-01	4.59e-01−1.16e+00	3518.22	2.84e-01	5.57e-02;9.71e-01	3914.5
**mixed carc**	With temporal signal	2.02E+00	1.64e+00−2.39e+00	3998.25	2.01e+00	1.65e+00;2.38e+00	3633.48

	Model	MCMC chains of 1e5 iterations			MCMC chains of 1e7 iterations		
		Mean substitution rate (μ)	95% CI	DIC	Mean substitution rate (μ)	95% CI	DIC
	No temporal signal	8.85E-01	6.13e-01−1.09e+00	3382.88	6.18e-01	3.80e-02;1.05e+00	4037.49
**mixed gamma**	With temporal signal	4.13E+00	3.11e+00−5.40e+00	4139.37	5.46e+00	3.44e+00;7.20e+00	3928.16
	No temporal signal	2.91E-01	2.00e-01−4.66e-01	4522.81	7.55e+01	4.34e+00;2.98e+02	4774.97
**strict gamma**	With temporal signal	1.37E+00	1.31e+00−1.45e+00	3704.52	1.23e+00	1.06e+00;1.36e+00	3706.46
	No temporal signal	7.77E-01	7.49e-01−8.06e-01	3860.67	2.56e-01	1.43e-01;3.63e-01	3875.9
**relaxed gamma**	With temporal signal	4.85E+00	3.52e+00−6.58e+00	4303.46	5.62e+00	4.26e+00;7.25e+00	4079.67
	No temporal signal	8.52E-01	6.24e-01−1.14e+00	4197.76	1.70e+00	1.69e-01;6.42e+00	4776.13

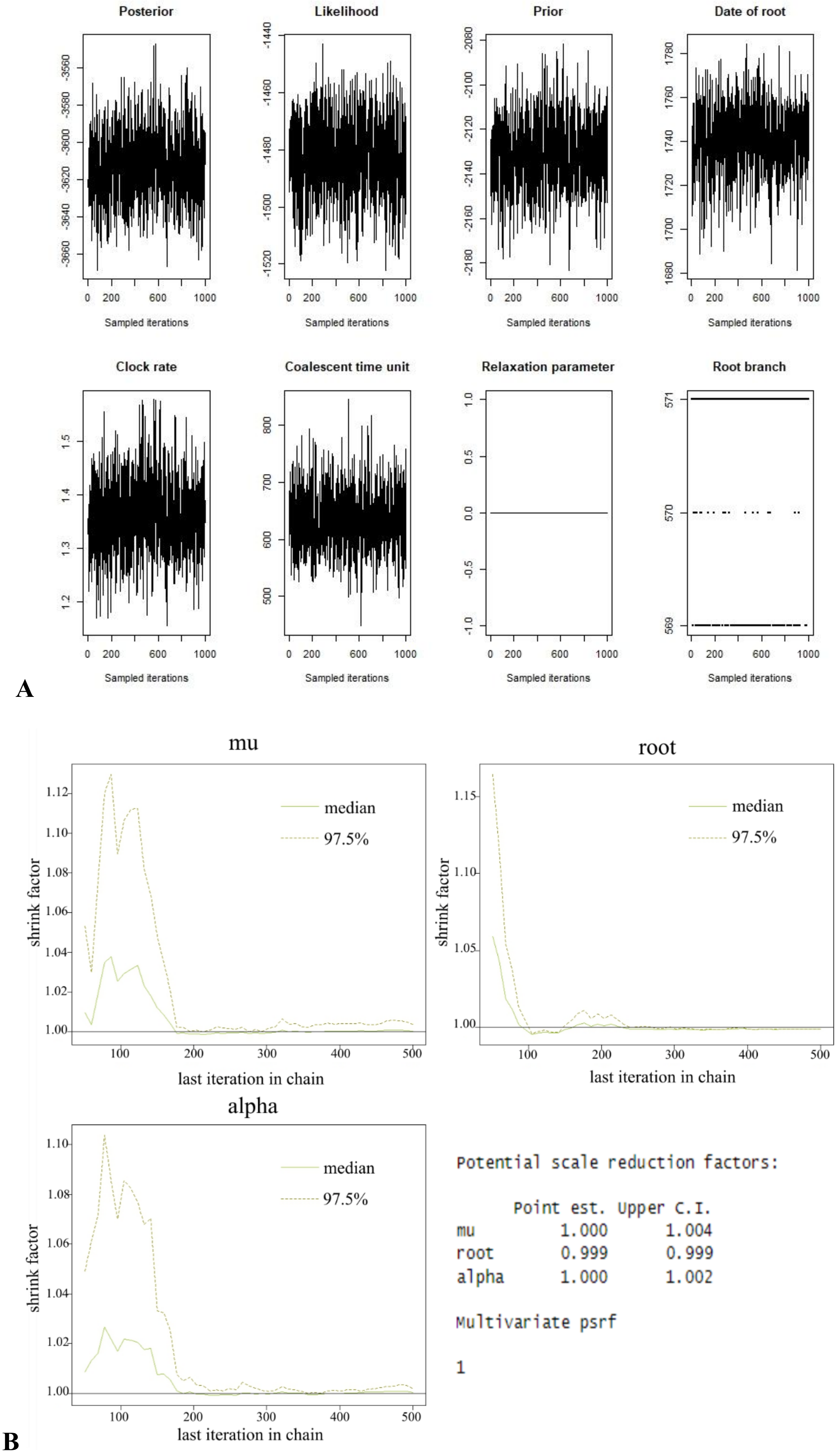

Supplementary Figure S1. *(A) Traces and (B) Gelman-Rubin convergence diagnostic of, respectively, one and three BactDating MCMC chains using Poisson model with 1e7 iterations.*

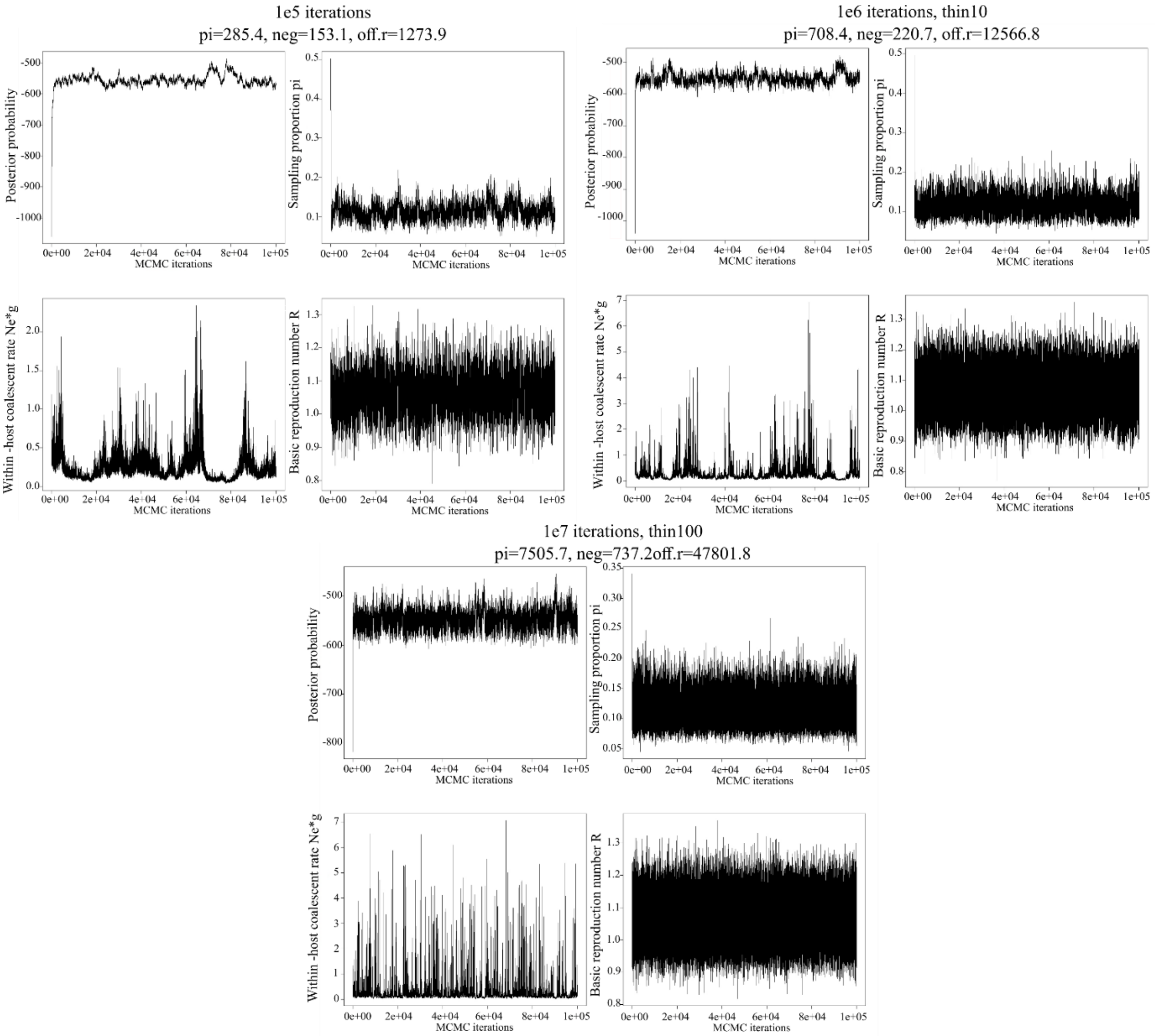

Supplementary Figure S2. *Traces of the TransPhylo MCMC chains using Poisson model with 1e5, 1e6 and 1e7 iterations. ESS values of parameters pi (probability of sampling an infected individual), off.r (parameter of the negative binomial distribution for offspring number) and neg (the within-host effective population size times generation duration) are shown for each MCMC chain.*
